# Prevalence of anelloviruses (TTV, TTMDV, and TTMV) in healthy blood donors and in patients infected with HBV or HCV in Qatar

**DOI:** 10.1186/s12985-016-0664-6

**Published:** 2016-12-28

**Authors:** Ahmed A. Al-Qahtani, Enas S. Alabsi, Raed AbuOdeh, Lukman Thalib, Gheyath K. Nasrallah

**Affiliations:** 1 Department of Infection and Immunity, Research Center, King Faisal Specialist Hospital and Research Center, Riyadh, Saudi Arabia; 2 Department of Microbiology and Immunology, Alfaisal University School of Medicine, Riyadh, Saudi Arabia; 3 Liver Disease Research Center, King Saud University, Riyadh, Saudi Arabia; 4 Department Health Sciences, College of Arts and Sciences, Qatar University, PO Box 2713, Doha, Qatar; 5 Biomedical Research Center, Qatar University, Doha, Qatar; 6 Department of Medical Laboratory Sciences, University of Sharjah, Sharjah, UAE

**Keywords:** Anelloviruses, HBV, HCV, PCR

## Abstract

**Background:**

Anelloviruses (TTV, TTMV, and TTMDV) have been associated with non A-G hepatitis. The goal of the current study was to estimate the prevalence of these anelloviruses in Qatar.

**Methods:**

A total of 607 blood samples (500 healthy donors, and 53 HBV-and 54 HCV-positive patients) representing different nationalities were tested for the presence of TTV, TTMV, and TTMDV DNA by nested PCR.

**Results:**

Prevalence rates for the three viruses were high in all studied groups, and exceeding 95% in the HBV group (for TTV and TTMDV). Infection with more than one type of viruses was common and significant in most of the positive patients (*p* < 0.05) and ranging from 55.4% for TTV/TTMV and TTMV/TTMDV co-infections in the healthy group, to 96.3% for TTV/TTMV co-infections in the HBV group. Further, and as with most previous studies, no significant association was found between anelloviruses infections and age, nationality, or gender (*p* > 0.05) albeit the detection of higher infection rates among females and Qatari subjects.

**Conclusion:**

This was the first published study to look at prevalence of Anellowviruses in the Middle East. High prevalence rates of the three viruses in all studied groups was noted. Further studies are needed to explore and compare the different genotypes of these viruses in the region.

## Background

Human infections with anelloviruses (small ssDNA viruses) are widely spread [1, 2]. A prominent member of the *Anelloviridae* family is the *Torque teno virus* (TTV), reported for the first time in 1997 in a Japanese patient with post-transfusion hepatitis of unknown etiology [3]. Subsequently (in 2000), another small DNA virus, designated *Torque teno mini virus* (TTMV), remotely similar to TTV was discovered [4]. In 2007 a third addition to the anellovirus genus was reported; the new virus had a genome of 3.2 kb (TTV and TTMV were around 3.8 and 2.8 kb, respectively), hence the designation *Torque teno midi virus* (TTMDV) [5]. The *Anelloviridae* family contains 12 genera; TTV, TTMV, and TTMDV belong to *Alphatorquevirus*, *Betatorquevirus*, and *Gammatorquevirus*, genera respectively [6].

The *Alphatorquevirus* genera (TTV) alone has 29 species. Much less is known about phylogenetic groups for TTMV and TTMDV [7]; TTMV ORF1 sequence comparison showed significant divergence, and complete genome analysis of TTMDV also shows sequences clustering [7].

The three viruses share a similar genome organization (UTR region, open reading frame region followed by short G/C rich region). Further, the three viruses are extremely divergent in the ORF regions both at the nucleotide and amino acid levels [6].

Furthermore, infection with anelloviruses can be highly common reaching a 90% prevalence rate [8]. Reports on the prevalence of TTMV in humans indicated high rates of infection in French [9], Brazilian [10, 11] and in Norwegian [12] blood donors and also in French hemodialysis patients (95%) [13]. Further, TTMV DNA has been detected in amniotic fluid, cord blood and breast milk [10, 14]. TTMV DNA has also been detected in cervical swabs of healthy women [15], women diagnosed with cervical cancers, peripheral blood mononuclear cells, feces and saliva [16].

TTMDV DNA was detected in plasma and saliva samples of French blood donors [17], in healthy and hepatitis C Italian serum samples [18], in healthy blood donors, hepatitis C patients and HIV patients in Iran [19], and in Korean chronic hepatitis patients and blood products [20]. TTMDV was also reported in Hungarian children with acute respiratory disease [21].

Since TTV was first detected in blood samples it was referred to as transfusion-transmitted (TT) virus [3]. Subsequent studies suggested the existence of other ways of TTV transmission including parenteral, sexual, vertical and others [22].

TTV has been detected in almost every human tissue type or body fluid, the newly discovered TTMV can be transmitted from mother to baby; similar virus sequences in mother and baby were detected in umbilical cord blood, breast milk, amniotic fluid, and sera of newborns [7].

Finally, viremic infections with multiple genotypes of any of the three viruses at any one time is not uncommon [12, 17, 23].

The aim of the current study was to investigate the rates of infection of TTV, TTMV, and TTMDV in Qatar, and to examine the association of these virus infections, if any, with HBV or HCV.

## Methods

### Ethical approval

Prior to samples collection from patients and blood donors, Institutional Review Board of Hamad Medical Corporation (HMC) (Protocol No.13422/13) and Qatar University (IRB No. QU-IRB289-A/14) approvals were obtained. Furthermore, because this research involves no risk to the participants, a waiver of all the consent requirements was obtained. The participants’ rights and welfare were not harmfully affected, because personal information related to participants was not claimed. Blood samples obtained from the blood bank and the hospital were anonymous. The only information collected related to the participants were nationality, sex, and age. Information concerning collected data was kept confidential.

### Blood samples collection

The samples that were used in this project to detect TTMDV and TTMV DNA were exactly the same ones we used in our previous TTV published project [8], and the data from it were used again in this study for comparison purposes between the three anelloviruses.

A total of 500 samples from healthy blood donors were used in the present study. These samples were collected from the blood donation unit during the period from June to August 2013. In addition, blood samples from 54 infected HBV to 53 HCV patients were also obtained from the diagnostic virology laboratory at the HMC.

### Plasma DNA extraction

DNA was extracted from 200 μl aliquots of plasma using a standard commercial kit for viral DNA extraction from body fluids (PureLink® Viral RNA/DNA Kits - Life Technologies; Grand Island, New York, USA).

### Whole genome amplification of DNA

Due to small sample volume, extracted DNA was amplified using REPLI-g Mini Kit (Catalog # 150025, Qiagen, USA) following manufacturer’s protocol. Amplified DNA was stored at −20 °C until use.

### Detection of TTMDV and TTMV

Both viruses were detected using nested PCR. For the first round PCR setup, the following components were used: one μl of whole genome amplified DNA, 10 pmol each of forward and reverse primers (universal primers) as described previously [24] and GoTaq Green Master Mix (Promega, Madison, WI, USA) in a reaction volume of 50 μl (μl). The mixture was preheated at 95°C for 5 min followed by 35 cycles with parameters as follows: 95°C for 30 s, 55°C for 30 s, 72°C for 30 s, and a final extension of 72 °C for 5 min. For the second round, two PCR reactions to detect TTMDV and TTMV were separately prepared. For the detection of TTMDV, the reaction mixture consisted of 5 μl of the amplification product of the first round, 15 pmol of NG795/NG796 primers [24] and GoTaq Green Master Mix with a total volume of 40 μl. The mixture was preheated at 95°C for 5 min followed by 30 cycles of 95°C for 20 s, 62°C for 20 s, 72°C for 20 s, and a final extension of 72°C for 5 min. The same conditions were used to detect TTMV except that the primers NG792/NG793/NG794 as sense primers and NG791 as antisense primer were used [24]. Fifteen μl of the amplified products were electrophoresed in 2.5% agarose gel, stained with GreenView Plus (GeneCopoeia, USA), and photographed under UV light.

### Statistical analysis

Data were analyzed using SPSS software. The chi-square test was used to compare proportions between the groups. Differences were considered to be statistically significant at was *p* < 0.05.

## Results

### Study population analysis

In the present study, a total of 607 plasma samples were screened for the presence of anellovirsuses (TTV, TTMDV, TTMV) DNA using nested PCR. The majority of samples were from healthy blood donors and non-Qatari male nationals. Age, gender and nationality distributions were not significantly different in healthy blood donors and HBV- or HCV-positive patients. Blood donors in Qatar were predominantly males, and so did those tested positive for HBV and HCV. It should be noted that the expatriate population makes up the vast majority of residents in Qatar with about only 16% of those residing in Qatar are Qatari nationals. Our study population reflects the demographic distribution in the population. In terms of age, it appears that the majority of the HBV patients were younger than 30 years whilst the majority of HCV patients were older than 50 years as was shown in Table 1.

**Table 1:** Characteristics of the subjects (*n* = 607)

		Healthy (*n* = 500) *n* (%)	HBV (*n* = 54) *n* (%)	HCV (*n* = 53) *n* (%)
Gender	Male	488 (97.6)	39 (72.2)	39 (73.6)
	Female	12 (2.4)	15 (27.8)	14 (26.4)
Nationality	Qatari	60 (12.0)	9 (16.7)	12 (22.6)
	Non-Qatari	440 (88.0)	45 (83.3)	41 (77.4)
Age (years)	<30	122 (24.4)	22 (40.7)	9 (17.0)
	31–40	227 (45.4)	18 (33.3)	9 (17.0)
	41–50	108 (21.6)	6 (11.1)	12 (22.6)
	>51	43 (8.6)	8 (14.8)	23 (43.4)
Age range (Average ± SD)	13 − 80 (36 ± 10.6)	19 − 74 (36 ± 9.3)	13 − 74 (34.9 ± 12.0)	15 − 80 (45 ± 14.9)

### Anelloviruses infection rates in the different populations

**Fig. 1:**
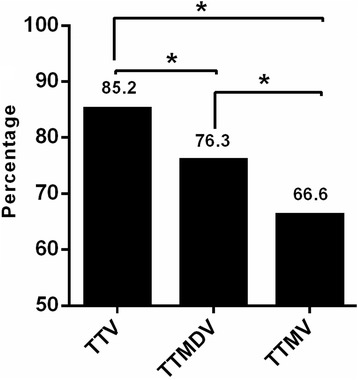
Prevalence of TTV, TTMDV, and TTMV in the overall study population (*n* = 607) * Significant difference (*p* < 0.05)

In order to determine the rate of infection of the three anelloviruses, plasma DNA was extracted from a total of 607 blood samples representing healthy blood donors (*n* = 500), HBV (*n* = 54), and HCV (*n* = 53) -infected patients residing in Qatar, and DNA of each virus was screened by nested PCR as described in the Methods. Figure 1 depicts the prevalence of TTV, TTMDV and TTMV DNA among the overall studied population. The overall prevalence of TTV DNA (85.2%) was significantly (*p* <0.001) higher than TTMDV (76.3%), and TTMV (66.6%). Further, TTV prevalence was significantly (*p* <0.001) higher among the healthy donor population than TTMDV and TTMV (Table 2).

**Table 2:** TTV, TTMV, and TTMDV distribution in study groups

	Healthy (*n* = 500) *n* (%)	HBV (*n* = 54) *n* (%)	*p*-value *	HCV (*n* = 53) *n* (%)	*p*-value**
TTV	417 (83.4)	52 (96.3)	<0.01	46 (86.8)	0.52
TTMDV	373 (74.6)	53 (98.1)	<0.001	47 (88.7)	0.02
TTMV	306 (61.2)	46 (85.2)	<0.001	44 (83.0)	<0.001
*** *p*-value	<0.001	0.04		0.69	

* Healthy vs HBV, ** Healthy vs HCV, *** TTV vs TTMDV vs TTMV

We also noticed that patients co-infected with HBV or HCV showed significantly (*p* <0.05) higher infection rates with anelloviruses than the healthy blood donors. The only exception was in the insignificant association between TTV and HCV infection. A closer look at Table 2 revealed that the highest prevalence of the three viruses was among the HBV followed by the HCV- infected patients.

### Analysis of mixed infections

Our data also showed that co-infections between the three anelloviruses was also very common (Table 3). The study found that 67.2% of healthy blood donors were infected with both TTV and TTMDV (*p* <0.001), and 55.4% were infected with TTV and TTMV or TTMDV and TTMV (*p* <0.001). The co-infection rates were significantly higher in blood donors (*p* < 0.001); there was no significant association between infections with any of TTV and TTMDV, TTV and TTMV or TTMDV and TTMV among HBV or HCV-infected patients.

**Table 3:** Association between TTV, TTMV, and TTMDV in different study populations

		TTMDV *n* (%)	*p*-value	TTMV *n* (%)	*p* -value
Healthy (*n* = 500)	TTV	336 (67.2)	<0.001	277 (55.4)	<0.001
	TTMDV	−	−	277 (55.4)	<0.001
HBV (*n* = 54)	TTV	46 (85.2)	0.14	52 (96.3)	0.03
	TTMDV	−	−	46 (85.2)	0.02
HCV (*n* = 53)	TTV	41 (77.4)	0.05	43 (81.1)	0.02
	TTMDV	−	−	39 (73.6)	0.33

The distribution of infection with TTV, TTMDV, or TTMDV among different nationalities, ages, and gender was shown in Table 4; there was no significant association between infection with TTV, TTMDV, or TTMDV and age or gender.

**Table 4:** TTV, TTMV, and TTMDV distribution in study groups (*n* = 607)

		Total (*n*)	TTV *n* (%)	*p*-value	TTMDV *n* (%)	*p* -value	TTMV *n* (%)	*p* -value
Gender	Male	566	47 (84.6)	0.11	428 (75.6)	0.10	375 (66.3)	0.34
	Female	41	38 (92.7)		35 (85.4)		29 (70.7)	
Nationality	Qatari	81	73 (90.1)	0.11	67 (82.7)	0.09	58 (71.6)	0.18
	Non-Qatari	526	444 (84.4)		396 (75.3)		346 (65.8)	
Age (Years)	<30	153	134 (87.6)	0.45	107 (69.9)	0.06	97 (63.4)	0.25
	31–40	254	217 (85.4)		204 (80.3)		171 (67.3)	
	41–50	126	102 (81.0)		92 (73.0)		80 (63.5)	
	>51	74	64 (86.5)		60 (81.1)		56 (75.7)	

Furthermore, since the expatriate population makes up the vast majority of residents in Qatar, we sought to study the distribution of the three anelloviruses in different nationalities residing in Qatar (Tables 2 and 5). As shown in Table 2, although the Qatari population had the highest prevalence of the three anelloviruses, the difference was not statistically significant.

**Table 5:** TTV, TTMV, and TTMDV distribution in different nationalities (*n* = 607)

	Qatar (=81) *n* (%)	Asia (*n* = 345) *n* (%)	Africa (*n* = 156) *n* (%)	Others (*n* = 25) *n* (%)	*p*-value
TTV	73 (90.1)	294 (85.2)	127 (81.4)	23 (92.0)	0.23
TTMDV	67 (82.7)	265 (76.8)	109 (69.9)	22 (88.0)	0.06
TTMV	58 (71.6)	223 (64.6)	105 (67.3)	18 (72.0)	0.60

Further, as shown in Table 5, there was no significant differences in the prevalence of TTV, TTMDV, and TTMV among the different ethnic or nationality groups residing in Qatar, which further suggests that nationalities or population origin has no effect on the prevalence of these viruses in Qatar.

## Discussion

Since their discovery, the three anelloviruses TTV, TTMV, and TTMDV shared some common features such as their detection in the blood of healthy donors and in HBV/HCV pationts. The presence of the viruses in non- A to G hepatitis patients, linked them to hepatitis. This probably justifies the number of studies, specially on TTV, that evaluated the prevalence of the virus in HBV and HCV blood samples compared to the limited number of studies in healthy individuals especially in the Middle East region. In a previous study [8] we reported our finding concerning the prevalence of TTV in healthy blood donors and HBV/HBC patients in Qatar. Therefore, the objective of this study was to evaluate the TTMV and TTMDV prevalence rates in healthy, as well as, HBV and HCV subjects in Qatar and compare the findings to those in the previous TTV study.

The current study was conducted on a total of 607 blood samples from healthy blood donors (*n* = 500), subjects with HBV (*n* = 54) or HBC (*n* = 53). Findings of the present study showed an overall high prevalence rates of the three viruses (Fig. 1); TTV prevalence rates (85.2%t) were significantly higher (*p* < 0.05) than the other two viruses (76.3 and 66.6% for TTMDV, and TTMV, respectively). A closer comparative look at the ditribution of the three viruses in the different study groups (healthy, HBV, and HCV) revealed interesting findings. Firstly, the incidences of the three viruses were significantly high in the HBV and HCV groups as compared to the healthy group (*p* < 0.05) (Table 2); prevalence rates were even highre in the HBV group (reaching 98% in the HBV group for TTMDV). Secondly, the prevalence rates of the three viruses were significantly different in the healthy (*p* < 0.001) and HBV (*p* < 0.04) groups but not in the HCV group (Table 2).

Further, most of the tested samples, regarless of the study group, yielded mixed infections; the highest association was seen in the HBV group between TTV and TTMV (96.3% of the samples); most of the associations were significant (*p* < 0.05) except for the mixed TTV/TTMDV infecitons in the HBV group and TTMDV/TTMV mixed infections in the HCV group (Table 3).

Prevalence rates for the three viruses were not significantly different in relation to gender, nationality or age (Table 4) (*p* > 0.05), albeit the slightly higher infection rates in females and Qatari nationals. A closer look at the overall distribution of the viruses in different ethnic backgrounds as compared to Qatari nationals revealed higher rates in Qataris and “others” compared to Asians and Africans (*p* > 0.05) (Table 5).

There is great prevalence variation of anelloviruses in different regions of the world. This variability can be explained in different ways; it is probably a true representation of the ubiquity of the viruses; it can be also attributed to the choice of DNA target region amplified and PCR primers used.

The high prevalence rates of anelloviruses viremias in this study, and other studies for that matter, can be also explained in part, by the ease of transmission of the virus.

Compared to TTV, much less is known about the prevalence rates of the TTMV and TTMDV in healthy individuals, as well as HBV and HBC patients. Earlier reports on the prevalence of TTMV in other regions indicated comperable infection rates in blood donors from France (76–77%) [9], Brazil (72–77%) [10, 11] and Norway (48%) [12]. Furthermore, lower prevalence rates were previously reported in healthy blood donors in Korea (41.3%) [25] and Iran (17%) [26].

With regards to TTMDV, the viremia frequency of the virus in healthy individuals was 14.5% in Iran [19], 34.5% in Korea [27], 20% in France [27], and 8.6% in Italian blood donors [18]. Those findings are much lower than the 74.6% reported in the current study in Qatar.

Due to the scarcity of data regarding the incidence of TTMV and TTMDV in HBV- or HCV-positive subjects, it became more difficult to make any comparative analysis. An earlier study in Iran reported incidence rate of 20% (5/25) and 48% (12/25) of TTMV in HBV and HCV subjects [26]. Further, Garcia-Alvarez et al. reported a very high incidence rate of TTV and TTMV in HIV/HCV-coinfected patients, which is higher than the 83% infection rate in HCV patients in the current study [28]. Also, in another study in Iran, researchers reported a TTMDV incidence rate of 80.5% (29/36) and 75% (3/4) in HIV/HCV and HIV/HCV/HBV coinfected patients, respectively [19], which was lower than the 88.7 and 98.1% TTMDV infection rates obtained in HCV and HBV infected patients in the current study (Table 2).

High frequencies of TTMV and TTMDV among HBV and HBC patients in comparison to low frequencies in healthy blood donors suggest common infection routes, highly compatible with these viruses, such as blood and sex.

Moreover, coinfection with multiple TT viruses seems to be a common event [28–30]. One explanation for the mixed infections with different anelloviruses seen in the current study and other studies would be that the viruses may have similar and probably synergystic infection cycles that help viruses from the same family better adapt to the environment. Another explanation would be that these viruses probably infect the same cell types permitting the viruses to favorably replicate in the same cells.

## Conclusions

In conclusion, this study showed a high prevalence (>75%) of the three anelloviruses (TTV, TTMDV, and TTMV) in the Middle East. Further studies are needed to explore and compare the different genotypes of these viruses in the region.
