# Erratum to: prevalence of anelloviruses (TTV, TTMDV, and TTMV) in healthy blood donors and in patients infected with HBV or HCV in Qatar

**DOI:** 10.1186/s12985-017-0791-8

**Published:** 2017-07-27

**Authors:** Ahmed A. Al-Qahtani, Enas S. Alabsi, Raed AbuOdeh, Lukman Thalib, Mohamed E. El Zowalaty, Gheyath K. Nasrallah

**Affiliations:** 1 Department of Infection and Immunity, Research Center, King Faisal Specialist Hospital and Research Center, Riyadh, Saudi Arabia; 2 Department of Microbiology and Immunology, Alfaisal University School of Medicine, Riyadh, Saudi Arabia; 3 Liver Disease Research Center, King Saud University, Riyadh, Saudi Arabia; 4 Department Health Sciences, College of Arts and Sciences, Qatar University, PO Box 2713, Doha, Qatar; 5 Biomedical Research Center, Qatar University, Doha, Qatar; 6 Department of Medical Laboratory Sciences, University of Sharjah, Sharjah, UAE; 7 Virology and Microbiology Research Laboratory & Antibiotic Research Unit, School of Health Sciences, University of KwaZulu Natal, University Rd, Westville Campus, Durban, 4000 South Africa

## Erratum

Following publication of this article [1] it has come to our attention that Dr. Mohamed El Zowalaty should have been listed as a contributing author having been involved in the data entry, figure construction, writing and revision of manuscript and table generation. The original version has been revised to reflect this.
